# Fast Data Generation for Training Deep-Learning 3D Reconstruction Approaches for Camera Arrays

**DOI:** 10.3390/jimaging10010007

**Published:** 2023-12-27

**Authors:** Théo Barrios, Stéphanie Prévost, Céline Loscos

**Affiliations:** LICIIS Laboratory, University of Reims Champagne-Ardenne, 51100 Reims, France; celine.loscos@univ-reims.fr

**Keywords:** 3D vision, training database, deep learning, 3D reconstruction

## Abstract

In the last decade, many neural network algorithms have been proposed to solve depth reconstruction. Our focus is on reconstruction from images captured by multi-camera arrays which are a grid of vertically and horizontally aligned cameras that are uniformly spaced. Training these networks using supervised learning requires data with ground truth. Existing datasets are simulating specific configurations. For example, they represent a fixed-size camera array or a fixed space between cameras. When the distance between cameras is small, the array is said to be with a short baseline. Light-field cameras, with a baseline of less than a centimeter, are for instance in this category. On the contrary, an array with large space between cameras is said to be of a wide baseline. In this paper, we present a purely virtual data generator to create large training datasets: this generator can adapt to any camera array configuration. Parameters are for instance the size (number of cameras) and the distance between two cameras. The generator creates virtual scenes by randomly selecting objects and textures and following user-defined parameters like the disparity range or image parameters (resolution, color space). Generated data are used only for the learning phase. They are unrealistic but can present concrete challenges for disparity reconstruction such as thin elements and the random assignment of textures to objects to avoid color bias. Our experiments focus on wide-baseline configuration which requires more datasets. We validate the generator by testing the generated datasets with known deep-learning approaches as well as depth reconstruction algorithms in order to validate them. The validation experiments have proven successful.

## 1. Introduction

The principle of photogrammetric 3D reconstruction is to recover the depth of a scene by exploiting the parallax existing on images acquired from different viewpoints. More precisely, this means matching pixels from one image with others (co-homologous pixels, i.e., projections of the same 3D point in images). The search space for co-homologous pixels [1] varies according to the structured (aligned, planar) or unstructured (free position) configuration of the cameras in the acquisition system. These variations have a decisive influence on the process of reconstructing a 3D scene from images. In this paper, we focus solely on 2D camera array configuration, where the principles of simplified epipolar geometry [2] can be applied. Thanks to them, the search space is reduced to a single line following the pixel grid of the image, i.e., vertical for vertically adjacent cameras and horizontal for horizontally adjacent cameras.

In this configuration, the depth computation becomes a disparity computation (i.e., the computation of an offset of a number of pixels separating the co-homologous pixels of 2 successive images in one of the horizontal or vertical axes). The use of deep neural networks for photogrammetric 3D reconstruction had a significant impact on improving state-of-the-art performance in terms of speed, accuracy, and robustness of reconstruction in stereo and light field configurations. However, they require training datasets, of more or less significant size depending on the camera configuration, usually including ground truth information. While reconstruction methods for light field cameras can be trained on a small number of scenes (state-of-the-art methods can be trained with a few dozen scenes), this is not the case for stereo and wide-baseline multi-view stereo configurations, which require a high number of training scenes (several thousand) to be efficient. The main reason for this is the wide range of correspondence search space. The light field configuration has a disparity of approximately 10 pixels, while the stereo and camera array configurations have a disparity range of around 200 pixels. The latter configurations, therefore, require a larger amount of data to train the network.

Some contributions have attempted to work around this problem by proposing deep neural network training without the need for ground-truth data, with either unsupervised [3,4] or self-supervised training [5]. Other works propose virtual datasets that have by construction more accurate ground truth data, and for some of them, more data [6,7]. However, a lot of these datasets only have a few dozen images and are thus more suited for method evaluation rather than training.

In this paper, we propose a dataset generator, i.e., to create a high number of scenes, and render them in the form of images and disparity maps, from a user-chosen set of models and textures. We show that our approach allows for a fast generation of a training dataset with enough variety to improve the results of deep learning methods for disparity estimation. We also demonstrate that the proposed dataset is best used for first-step training before fine-tuning is performed with a state-of-the-art dataset.

After a review of different types of available state-of-the-art datasets in Section 2, we present our highly configurable generator and describe our training dataset and the protocol for our experiments in Section 3. The experiments in Section 4 compare the use of our dataset versus Li et al.’s dataset [7] for training. They highlight the relevance of our training dataset, and hence such a generator, by comparing use cases with two deep learning reconstruction methods [7,8], firstly, as a single source, secondly as a primary, and finally as a fine-tuning dataset. We conclude and address future work in Section 5.

## 2. Related Work

In this section, we distinguish three types of available data to review the state-of-the-art datasets/generators. The first is real data, where images are recorded through sensors, such as cameras, possibly with ground truth using depth cameras, or Lidar sensors. The second is hand-made virtual data, i.e., scenes that are manually created and rendered with 3D modeling software but where scene conception and lighting are decided by a human being. The third type is procedurally generated data, where scene conception is decided by an algorithm.

In Table 1, we summarize the features of discussed training datasets in this section. For a more extensive review, please refer to [9].

### 2.1. Real Datasets

Most of the real scene datasets were made for testing purposes rather than training. Before the emergence of machine learning techniques in stereoscopic reconstruction methods, real scenes were provided as benchmarks for method evaluations, as for example by Scharstein et al. [10,11]. More recently, several benchmarks were proposed for stereo reconstruction and unstructured multi-view stereo reconstruction, made of real scenes associated with their ground truth data, expressed in the form of a disparity or depth map [11,12,13]. Early deep neural network methods, such as [18], were trainable on the small number of scenes, offered by these datasets (around 20 scenes).

In 2015, Menze and Geiger [12] also proposed a set of 200 real training scenes for the purpose of stereo disparity reconstruction on car-embedded cameras. The scenes are exclusively driving scenes and serve the purpose of autonomous driving.

However, using real data involves handling the properties and imperfections of physical image sensors (optical and color distortions). Correspondingly, when depth is captured, it also means dealing with the inaccuracy of the depth sensor (noise), and sometimes its inability to provide ground truth values in certain areas (highly reflective, absorptive and transparent area, etc.). Moreover, due to their nature and size, none of these real datasets are used as standalone training datasets by current deep neural network methods. Nevertheless, the datasets can be also used for network fine-tuning, i.e., for adapting the weights of a pre-trained neural network to a specific context.

### 2.2. Hand-Made Virtual Datasets

Virtual datasets allow to have precise and complete data with ground truth. In the context of light field disparity reconstruction, Honauer et al. [14] proposed a benchmark and a hand-made training dataset with 25 scenes. This low number of scenes, compared to other configurations, is enough to train state-of-the-art methods for this configuration. Li et al. [7] proposed a training and a testing dataset for a 9 × 9 wide-baseline camera array with a disparity range of 50. The testing dataset is composed of 12 virtual hand-crafted scenes and the training dataset also contains eight hand-crafted scenes.

While most of these proposed datasets have very few scenes, some efforts were made in improving the scene variety by proposing datasets based on image sequences of animated scenes instead of still scenes [6,15]. This allows for the creation of a higher number of scenes than with hand-crafting scenes, within the same time span. However, the scenes generated by this method do not increase the variety of objects in the dataset.

### 2.3. Procedurally Generated Datasets

Procedurally generated scenes can be used to have a large amount of data, without the need for time-consuming human design. For the stereo configuration, Dosovitskiy et al. [16] proposed a training dataset with various chair models that are randomly positioned. Mayer et al. [6] proposed training and testing datasets with more variety in models based on the ShapeNet [19] taxonomy. Furthermore, textures for this dataset are randomized based on various existing and also procedurally generated images.

For camera arrays, Li et al. [7] proposed a similar process for generating a training dataset with a nearly photo-realistic rendering. This dataset contains 345 scenes, with images taken by a 9 × 9 camera array. While these images are very high quality, the relatively small number of scenes makes it only practical for training lightweight neural networks. The disparity range is set at 50 pixels disparity range. However, this range can be extended to 200 pixels if you consider the dataset as a 3 × 3 camera array, by taking the images on every fourth row and column. This dataset contains scenes with a realistic rendering, although, they are in small numbers and thus are only efficient for training lightweight neural networks—around 2 M weights for Li et al.

In summary, the state of the art lacks large datasets when it comes to 3D wide-baseline camera array reconstruction, and even more so when it comes to network training, as many do not have the necessary ground truth and/or do not have a sufficient quantity of data. Existing deep neural network methods rely on training on datasets of relatively small scale and thus need to adapt to these small scale datasets, limiting their efficiency. We thus propose a way to generate data suitable for training more heavyweight and data-sensitive neural networks.

## 3. Materials and Methods

### 3.1. Virtual Data Generator

#### 3.1.1. Principle

The goal of our data generator is to be able to: (i) quickly generate a large number of training scenes, with a great variety, for a two-dimensional camera array of a user-defined size; (ii) render and save these scenes as different data (RGB, disparity) to provide a dataset useful for 3D reconstruction methods. To be used in deep neural network supervised training, these scenes must also have associated disparity ground truth. For this, the principle is to randomly associate 3D models with a texture each, and randomly position them in order to render scenes taken from a virtual N×M camera array with a regular baseline, equal in the horizontal and vertical direction. The random selection of models ensures diversity of scene content, while the random assignment of textures to objects ensures that the method will not learn to recognize an object by its color, like green grass, blue sky, etc., thus avoiding a shape-color association bias. Similarly, to avoid shape-object association bias, we apply random scaling to each object on each axis.

**Figure 1:**
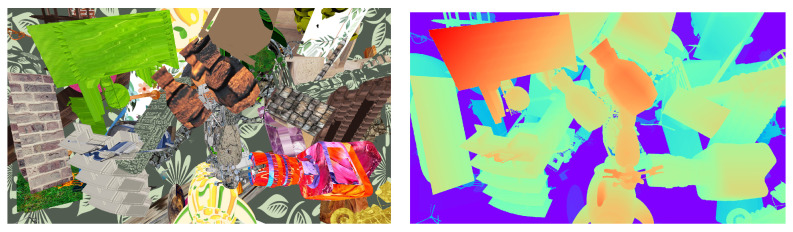
Example of our generator output. An RGB rendering image of one scene (**left**) with its disparity map (**right**) encoded on three RGB channels (see Section 3.1.3).

Although the generated images are non-realistic, and quite unintelligible for a human being, when taken as a whole as illustrated in Figure 1, their local geometry mimics the variety of shapes and colors that can be found in real scenarios. It is thus possible to train deep neural networks on such scenes.

#### 3.1.2. Parameters

Our proposed dataset generator allows for several types of parameters to be configured through its configuration file:

– Camera array configuration.

The number of cameras on each row and column (*cam_grid_row*, *cam_grid_Col*), as well as the baseline (*grid_spacing_row*, *grid_spacing_col*), i.e., the space between two adjacent cameras can be configured, one for each axis. This is one of the main factors in the disparity range of the created dataset. The virtual camera array can also be selected in off-axis or parallel disposition with the focus point parameter (*focusPoint*), although this paper focuses on the parallel disposition. When the focus point is set to 0, the cameras are positioned in parallel, otherwise, their vision pyramid is off-center to focus on it.

– Camera parameters.

The configuration is the same for every camera on the array. The configuration of their intrinsic matrix is realized with the following parameters: image resolution in pixel (*width_pixel*, *height_pixel*), *near* and *far* parameters. The vertical field of view (*fov*) can also be parameterized allowing for datasets from several types of cameras. As their positions in the camera array are constrained, we do not propose extrinsic parameter selection. The extrinsic matrix is fixed as the identity matrix since objects will be placed based on the camera field of view. It is modified according to the camera’s position in the grid. In addition, for future work and the adaptability of our generator, we already integrated the camera exposures (*exposures*). This will be useful for multi-exposed recording in a High Dynamic Range reconstruction context. If several values are entered, each viewpoint is rendered once with each exposure value.

The parameters in these two sections (Camera array configuration and Camera parameters) are used to generate the capture system of our generator (see line 4 of Algorithm 1).

**Algorithm 1** CameraArrayDatasetGenerator ()          ▹*A function for generating a dataset with ground truth from a camera Array respecting the parameters described in the user configuration file***Input:**     cfgFile                                              ▹ *configuration file***Output:**  *cam_grid_row*×*cam_grid_col* RGB images with their disparity maps (see Table 2) 
1:*cfg*   = *cfgFile* contents2:*models* = Load the models from the *model* file folder3:*texs*     = Load textures from the *texture* file folder4:*camArray* = Generate the camera arrays, initialized with the *camera parameters* and *camera array configuration* defined by the user in *cfgFile*5:Create the OpenGL Context with a size set at (*cfg.width_pixel*, *cfg.height_pixel*)6:**for** nbreCaptureToDo = cfg.number_of_frame_to_render;7:      nbreCaptureToDo > 0; nbreCaptureToDo- = 1 **do**8:  populateScene()9:  **for each** *cam* from *camArray* **do**10:    Render the view from *cam* with its disparity map and save the both in the *cfg.output_dir* folder11:   **end for**12:**end for**

– Scene configuration.

The major part of the configuration is on the scene. The minimum and maximum distances of objects can be set (*object_range*). However, these minimum and maximum values are not hard limits, as they are only used to position the object centers themselves. Part of the objects can still be in front of the minimum distance or behind the maximum distance. As a side-effect, some of the scenes generated do not conform to the desired maximum disparity. Other configuration parts are the different numbers of models (*n_models*) and textures (*n_textures*) loaded and how many times a given model is loaded with a different texture on a scene (*n_textures*). Finally, we propose to set the probability of hiding each object on each new scene (*visible*). The probability can be set in a range of probabilities so that some scenes are more or less full than others. Lastly, the user can set the number of generated scenes (*number_of_frame_to_render*). The Algorithm 2 gives step-by-step the construction of one randomly generated scene. In our model folder, we put only the required untextured models for the training. Random selection from this folder is not needed as we process models iteratively. This position is reflected in the Algorithm 2.

**Algorithm 2** populateScene ()  ▹*A function which randomly insert some randomly textured and distorted objects. The function random(x,y) generates a random value between x and y with a uniform distribution.***Input:**   *cfg*                                                ▹*configuration Structure***Input:**   *models*                                           ▹*set of the loaded models***Input:**   *texs*                                              ▹*set of the loaded textures*
1:  
proba = random(cfg.visible[0], cfg.visible[1]) 2:  **for** (i = 0; i < *cfg.n_models*; i++) **do**3:    **for** (j = 0; j < *cfg.n_textures*; j++) **do**4:        
model = Clone of *models[i]*5:        
tex = Random texture selection from *texs*6:        
dispZMin = $MIN({cfg.object\_range[0]}^{cfg.rep},{cfg.object\_range[1]}^{cfg.rep})$
7:        
dispZMax = $MAX({cfg.object\_range[0]}^{cfg.rep},{cfg.object\_range[1]}^{cfg.rep})$
8:        **if** (random(0,1) − proba ) > 0 **then**9:          zpos = random( dispZMin , dispZMax )10:        Translate the model on the z-axis by $zpos^{\frac{1}{cfg.rep}}$
11:         **else**12:              Hide the model13:         **end if**14:         Randomly translate of model on the x-axis and y-axis to place it in the frustum of the camera array15:         Randomly scale the model on each axis16:         Randomly rotate of model on each axis 17:         Add model to the 3D scene18:     **end for**19:**end for**

– Output.

Each rendered output (an RGB image with its disparity map) is saved in the defined output directory (*output_dir*).

#### 3.1.3. Implementation Details

We chose to implement our dataset generator as a webGL application using html and javascript, with the electron API [20] and threeJS [21]. Indeed, on the one hand, ThreeJs is a well-known javascript 3D library in the computer graphic world, which has also the advantage of already having a large number of mesh loaders (obj, ply, fbx, gltf, etc.). On the other hand, the web nature of this application allows it to be cross-platform and makes it easy for others to reuse.

Meshes and textures are each in their own directory, which must contain at least two items. Given the nature of our mesh data, we are currently only using the obj loader, but integrating the other loaders should not pose any problems.

The output data (RGB image and disparity map) is rendered from each camera of the array, using OpenGL rasterization [22] without incorporating any complex lighting effects (no shadow, no transparency, etc.). We generate the color rendering and the disparity map with two shader passes. We compute the depth as explained in [23] and we deduce from it the disparity value with the properties of the cameras in the array. This disparity value δ is instantly encoded in the shader as described in the following storage section.

The Algorithms 1 and 2 describe the global pipeline and main steps.

– Storage.

We save images and disparity maps as PNG images. Files are named as follows: {*tag*}{*type*}{*position*}_{*exp*}.png where:

- *tag* is a 21 alphanumeric scene label, randomly generated each time a new scene is rendered. This label has around $4.8\times 10^{32}$ different possibilities.
- *type* is either *rgb* for pictures or *depth* for disparity maps.
- *position* is a single number identifying the position of the view on the array. It identifies the view in a top-to-bottom, left-to-right order, i.e., if the position is (i,j) on a cam_grid_row×cam_grid_col array, the *position* number will be i.cam_grid_col+j .
- *exp* is the exposure factor, a higher number means brighter images, and a lower number means darker images. The *exp* value is the exposure value for RGB images and 0 for ground truth disparity maps (file with *type* = *depth*).

The disparity values are encoded in a 32-bit fixed-point precision format, with possible values ranging from 0 to 8192. The disparity is encoded using the four channels of the image:

- Red channel encoding the coarsest part of disparity with a disparity step of 32.
- Green channel encoding disparity with a step that is 256 times smaller, i.e., $\frac{1}{8}$ .
- Blue channel encoding disparity with a step of $\frac{1}{2048}$ .
- Alpha channel encoding disparity with a step of $\frac{1}{524,288}$ .

### 3.2. Creation of the Dataset

We propose a Full HD dataset for deep neural network training. It is taken from a 5 × 5 camera array in a parallel disposition. Image sizes are 1920 × 1080. We set the cameras *near* and *far* at 0.1 m and 1000 m, respectively, and the field of view at 60 degrees.

For models and textures, we took 51 models from the ShapeNet taxonomy [19], each from a different semantic class, and 100 texture-like images from the Pixabay [24]. In each scene, models are loaded three times with a random texture. Each textured model has a probability to be hidden varying between 30 and 60%.

Objects are positioned in a random place within the field of view of the camera array and at a distance between 2 and 500 m. To smooth disparity repartition the positioning is not uniform but based on the distance. The likelihood of an object being put at a given distance is $\frac{1}{\sqrt{x}}$ with *x* the distance to the camera array center. This means that objects are more likely to be put closer to the camera array than further. Combined with our rescaling making objects that are bigger, gives a smoother distribution.

The baseline for the camera array is set to 0.2 m for the horizontal and vertical axis. This gives this camera array a disparity range of 128 (from 0 to 128). We rendered 4000 images and the output datasets have a size of 185 GB and 526 GB, respectively. From this dataset, we remove the scenes with disparity outside the target range, which leaves us with 3978 scenes. The Table 2 summarizes the parameter settings. Since we do not experiment on multi-exposed camera arrays, the exposure value is always set to 1 in our experiments.

The generated dataset, which is also the one used in our experiments and following the configuration given in this article, is freely available to the community (see Data Availability Statement).

### 3.3. Protocol for Experiments

With the proposed camera array, we conduct experiments on a 3 × 3 configuration by taking views on every other row and column of our 5 × 5 dataset. For the training dataset, the disparity range is 0–256 in the 3 × 3 configuration. For experiments, we compare the results obtained by two deep neural network methods from Li et al. [7] and Barrios et al. [8]. It should be noted that our aim is not to compare these methods with each other, but only to compare the contribution of our dataset to these methods, without or with a fine-tuning.

– Considered training datasets

As shown in the related work section, no dataset with ground-truth exists in the literature suitable for training heavyweight and data-sensitive neural networks, in order to estimate disparities within a context of a wide-baseline camera array. As illustrated in the Table 1, only the Li et al. [7] dataset has ground truth, even though it was not originally designed for use in a wide-baseline scenario. The considered training dataset thus are the datasets described in this paper and the one proposed by Li et al. in [7]. Networks are trained with the same amount of iterations on either dataset. We then compare the results obtained by methods on Li et al.’s testing dataset, for the 3 × 3 configurations. We also propose experiments on training with one of these two datasets and refinement with the other. The refinement part consists of 10k additional iterations, with a learning rate fixed at 10 ${\;}^{-5}$ throughout the process for every network and dataset considered.

– Test metrics

For comparison, we use the metrics *bad x* that are used by Li et al. in their paper and also on the Middlebury Stereo website [25]. The metric *bad x* is the percentage of pixels for which the absolute error, when the resulting disparity is compared to the ground truth, is greater than *x*.

For comparison to ground truth, we use the testing dataset proposed by Li et al. in [7]. This dataset is originally a 9 × 9 testing dataset with a disparity range of 50 (from 0 to 50). For our experiments, we will use this dataset with a 3 × 3 array by using one in every four columns. The disparity ranges will thus be 200 for the 3 × 3 configuration. Due to the different configurations and disparity ranges, we adapt the metrics used by the Middlebury stereo benchmark [25]. We use for the 3 × 3 configuration, bad 0.5, 1, 2, and 4, similar to the main metrics on the reference.

– Tested methods

Two deep neural network methods are tested. The first one, proposed by Li et al. in [7], is a lightweight neural network with less than 2 M weights. It computes a disparity map mainly through convolution neural networks following the classical structure of disparity inference with deep neural networks [9].

The second tested method, proposed by Barrios et al. [8], is a neural network that computes disparity maps in two parts, following a similar structure. The first part computes a downsampled disparity map and the second step computes a residual disparity map from low to high resolution. The network has 5 M weights in total, with the second step counting more than 4 M weights.

These networks are trained on each dataset with the number of iterations or training time indicated on their respective papers, i.e., 1.5 M iterations for Barrios et al. [8] and 250 k iterations corresponding to the training time indicated on the paper [7].

## 4. Results and Discussion

### 4.1. General Results on the 3 × 3 Configuration

The results with the proposed metrics are shown in Table 3 for the 3 × 3 configuration. The results shown are the average results of the training performed.

**Figure 2:**
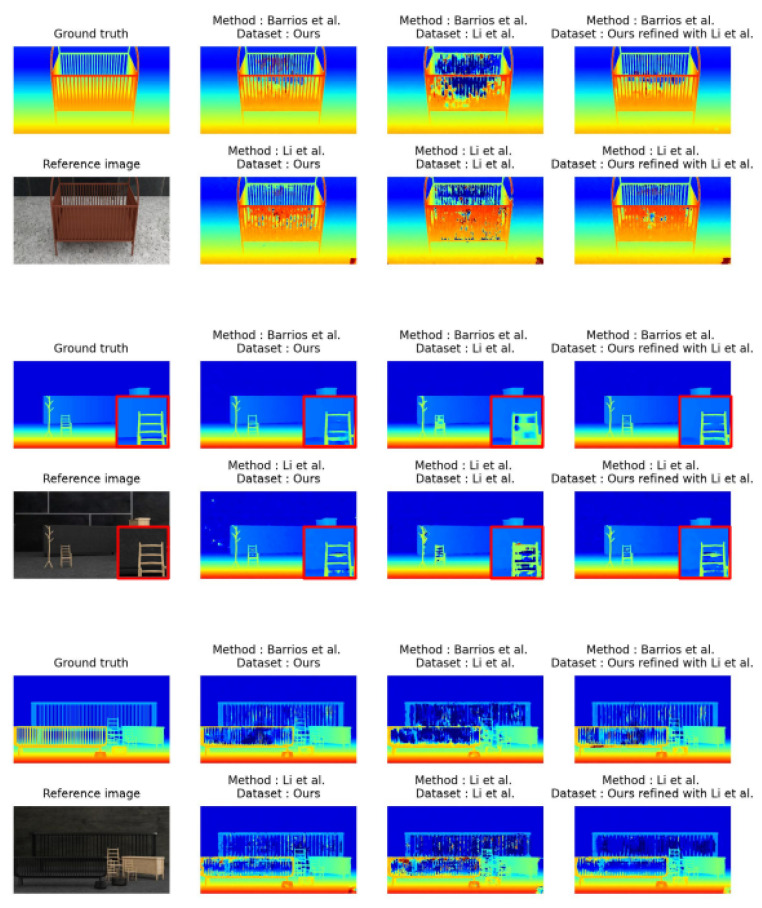
Difference of disparity maps between networks trained with ours and Li et al.’s with examples of images containing thin elements, taken from Li et al.’s [7] and Barrios et al.’s [8] test dataset. The red squares are zooms of a detailed part of the image.

On the 3 × 3 configuration, the results show that the use of our training dataset instead of that of Li et al. strongly improves results for the bad 2 and bad 4 metrics for both considered neural networks. For example, with Li et al.’s method, the bad 4 metric is 5.85 when trained with their dataset and 4.60 when trained with ours. While this 3 × 3 configuration is not optimal for Li et al., it remains a possible configuration for their method and our training dataset shows improvement. With the network from Barrios et al. using our training dataset improves the results from 5.75 to 3.59 for bad 4. With higher tolerance thresholds, these metrics can identify the outliers in reconstruction. Since a lower error rate means greater robustness, we can, therefore, conclude that using our training dataset instead of Li et al.’s significantly improves the robustness of deep neural networks. This is especially visible on images with thin elements, for example, on the image shown in Figure 2. In this figure, the thin elements are correctly reconstructed with a network trained with our dataset when it is not with networks trained on the dataset of [7]. This can be seen in the third column of the figure, where bars are more efficiently reconstructed when our training dataset is used.

**Figure 3:**
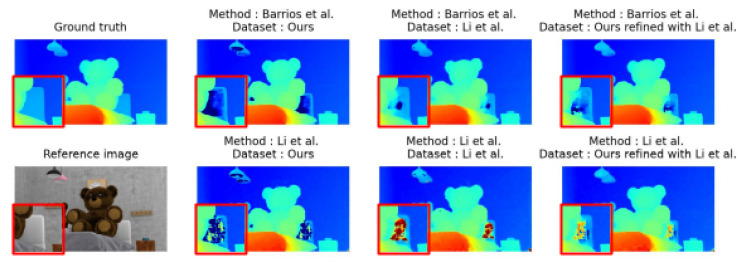
Difference of disparity maps between networks trained with ours and Li et al.’s with an example image some bright untextured elements. Images are taken from Li et al.’s [7] and Barrios et al.’s [8] test dataset. The red squares are zooms of a detailed part of the image.

When considering fine precision (bad 0.5), the results are different depending on the method that is considered. While training with our dataset improves the results obtained with the network from Barrios et al. [8] (with a bad 0.5 of 14.96 versus 16.60), they degrade the results with Li et al. (with 25.40 versus 23.95). This puts forward the main limitation of our dataset. As we chose rasterization as our method of rendering, some low textures and light effects are not taken into account. Our training dataset thus underperforms, even compared to the one in [7] when images contain low texture and light effects, as can be seen in Figure 3. The zone below the teddy arm on the right is not reconstructed correctly when the methods are trained with our dataset.

Moreover, optimal results are obtained when the network trained with our training dataset is fine-tuned with Li et al.’s dataset. This fine-tuning results in a better reconstruction rate than without fine-tuning regardless of the network, the training dataset, or the bad metric considered. These results are shown in the last column in Figure 2 and Figure 3. For each scene presented, detailed numerical results, shown in Table 4, confirm the conclusion but also indicate that on some views, results can be degraded by the fine-tuning step.

As shown in detail in Table 5, the fine-tuning process improves results on some pixels and degrades them on others. For example, Li et al.’s neural network using our dataset in initial training with a fine-tuning with the dataset of Li et al. (see Table 5), allows 9.92% and 7.21% of pixels to go from bad 1 to bad 0.5 and vice versa. Those values are 4.13% and 2.50%, respectively, for the method of Barrios et al. We can, however, note that for both neural networks considered the amount of pixels improved is higher than the amount of pixels degraded (15.82% vs. 11.15% in the method of Li et al. and 7.71% vs. 5.63% for the method of Barrios et al.), whether this is in total (on the first column of the tables) or when considering evolution from any two categories (considering two opposed categories, e.g., from errors between 0.5 and 1 to error smaller than 0.5 compared with the inverse evolution).

However, when the training and fine-tuning datasets are swapped, i.e., training is conducted with the dataset from Li et al. [7] and fine-tuning with our dataset, the results are significantly degraded. The third rows of each section of Table 3 show that results with this choice of training are worse than even non-fine-tuned training. We can thus conclude that our training dataset is not relevant for fine-tuning purposes. As the results are significantly worse with this reversed training, we do not propose a detailed analysis of the evolution as we did in the previous case.

### 4.2. Comparison with Data-Sensitive Networks

When considering solely the network from Barrios et al. [8], two things must be considered. First, when comparing training with either our dataset or Li et al.’s alone, Table 3 shows that using our dataset gives better results in every metric, whether it considers fine reconstruction or outliers. This is mostly due to the refinement step that is more data sensitive than the network from Li et al. and thus is not efficiently trained by Li et al.’s smaller-scale training dataset. This is also visible with the results in Table 6. When we only consider the downsampled disparity map of Barrios et al. [8] that was computed with a very lightweight network that has an overall structure similar to the one in [7], we observe the same behavior [7], i.e., better robustness but less overall precision when trained with our dataset compared to Li et al.’s.

Second, fine-tuning is less efficient when conducted on the refinement step of this method, as proved by the results obtained by turning on or off fine-tuning on the last part of the network in Table 7.

## 5. Conclusions and Future Work

We introduced a dataset generator to automatically compose scenes and render them as a set of images and disparity maps with a large variety from a set of user-defined models and textures. The scenes that we generate are nowhere near realistic in terms of color and their composition (layout of objects). Nevertheless, they present geometric challenges that are found in realistic scenes and avoid any shape-color association bias. As we opted for the very fast but limited rasterization render method, some light effects are not present in our dataset and methods trained with it cannot process them correctly. However, we showed that a short fine-tuning step on a smaller dataset that does take these light effects into account not only resolves this problem but obtains overall more stable results.

Future work includes testing different disparity ranges, from the very short disparity range as in lightfield configuration to wider disparity ranges, like what was proposed in this work. The objective would be to assert the amount of data required to train methods depending on the target disparity range. Another future work possibility is to find a compromise between the speed of rendering and its quality and accounting for specific light effects by changing the rendering engine to more modern engines that can provide fast rendering with a higher visual quality, such as Unreal Engine [26] or NVIDIA Omniverse [27]. In addition, it would also be interesting to extend our experiments by using a testing dataset consisting of real data and ground truths obtained by LIDAR technology.

## Figures and Tables

**Table 1:** Summary of discussed training datasets and ours. MVS: Unstructured multi-view stereo, LF: light field, #: number.

Reference	Nature	# Cameras	Structure	# Captures	Resolution	with GT
Mayer et al. [6]	both	2	stereo	≈25 k (200 real)	960 × 540	✓
Li et al. [7]	virtual	81	9 × 9 array	353	512 × 512	✓
Scharstein et al. [10]	real	2	stereo	6	512 × 384	✓
Scharstein et al. [11]	real	2	stereo	33	2864 × 1924	✓
Menze et al. [12]	real	2	stereo	400	1242 × 375	✓
Schops et al. [13]	real	4	MVS	38	6048 × 4032	✓
Honauer et al. [14]	virtual	81	9 × 9 LF	28	512 × 512	✓
Butler et al. [15]	virtual	2	stereo	1628	1024 × 436	✓
Dosovitskiy et al. [16]	virtual	2	stereo	≈22k	1024 × 768	✓
Sabater et al. [17]	real	16	camera array	12	2048 × 1088	×
Ours (for experiments)	virtual	25	5 × 5 array	3978	1920 × 1080	✓

**Table 2:** Parameter list with their name in the configuration file and their value for the experiment.

Parameter	Name in Configuration File	Experiment Value
camera array configuration		
nb Camera in a Row	cam_grid_row	5
nb Camera in a Column	cam_grid_col	5
baseline in meters in row and column	grid_spacing{_row, _col}	0.2
camera focus point (if off-center)	focusPoint	0
camera parameters		
image resolution in width	width_pixel	1920
image resolution in height	height_pixel	1080
Z near distance in meters	near	0.1
Z far distance in meters	far	1000
vertical field of view	fov	60
Camera exposures	exposures	[1.0]
Scene configuration		
Minimum and Maximum distances between object and camera array center (in meters)	object_range	[2, 500]
Number of different models loaded in the scene	n_models	51
number of repetition of model in a scene	n_textures	3
rate of hidden	visible	[0.3, 0.6]
scene number	number_of_frame_to_render	4000
Number of different textures in the directory		108

**Table 3:** Comparison of results between training with either our dataset and the one proposed in Li et al.’s work [7]. “Bad x” metrics represent the percentage of pixels for which the difference to ground truth is higher than x. Lower is better. Bold values have the lowest errors for their respective method and metric.

Method	Training Dataset	Bad 0.5	Bad 1	Bad 2	Bad 4
Results with a 3 × 3 configuration					
Li et al. [7]	Li et al. [7]	23.95	12.58	7.70	5.85
	Ours	25.40	11.49	6.46	4.60
	[7] fine-tuned with ours	27.27	14.95	10.14	7.74
	Ours fine-tuned with [7]	**22.90**	**10.28**	**5.78**	**3.96**
Barrios et al. [8]	Li et al. [7]	16.60	11.46	8.22	5.75
	Ours	14.96	8.27	5.33	3.59
	[7] fine-tuned with ours	20.14	12.78	9.19	6.79
	Ours fine-tuned with [7]	**12.23**	**7.51**	**4.60**	**2.98**

**Table 4:** Comparison of results between training with either our dataset and the one proposed in Li et al.’s work [7] on some specific views. “Bad x” metrics represent the percentage of pixels for which the difference to ground truth is higher than x. Lower is better. Bold values have the lowest errors for their respective method and metric.

Method	Training Dataset	Bad 0.5	Bad 1	Bad 2	Bad 4
Results for view #1, “Cot”					
Li et al. [7]	Li et al. [7]	32.52	20.56	15.95	13.73
	Ours	26.72	14.07	9.23	**7.00**
	[7] fine-tuned with ours	29.28	19.14	15.45	13.32
	Ours fine-tuned with [7]	**29.47**	**15.14**	**10.13**	7.88
Barrios et al. [8]	Li et al. [7]	32.01	26.77	22.73	18.97
	Ours	21.96	14.67	10.01	6.86
	[7] fine-tuned with ours	33.10	27.94	24.58	20.96
	Ours fine-tuned with [7]	**21.51**	**13.35**	**8.10**	**5.20**
Results for view #2, “Furniture”					
Li et al. [7]	Li et al. [7]	12.72	5.59	2.97	2.14
	Ours	24.77	7.84	2.04	1.17
	[7] fine-tuned with ours	30.40	16.40	11.42	9.07
	Ours fine-tuned with [7]	**16.53**	**5.08**	**1.96**	**1.10**
Barrios et al. [8]	Li et al. [7]	7.10	3.93	2.47	1.52
	Ours	11.41	2.59	1.31	0.66
	[7] fine-tuned with ours	10.85	4.97	2.88	1.82
	Ours fine-tuned with [7]	**5.56**	**2.43**	**1.24**	**0.63**
Results for view #3, “Sidebars”					
Li et al. [7]	Li et al. [7]	38.98	31.84	26.46	22.87
	Ours	38.19	26.22	19.65	15.74
	[7] fine-tuned with ours	43.85	35.44	30.06	26.06
	Ours fine-tuned with [7]	**35.56**	**26.17**	**19.56**	**15.35**
Barrios et al. [8]	Li et al. [7]	42.08	36.69	30.97	24.75
	Ours	36.65	28.02	22.20	17.44
	[7] fine-tuned with ours	45.21	39.40	35.47	30.93
	Ours fine-tuned with [7]	**34.21**	**26.98**	**20.78**	**15.96**
Results for view #4, “Teddy Bears”					
Li et al. [7]	Li et al. [7]	22.98	8.23	3.85	2.83
	Ours	**21.38**	**6.37**	3.54	2.88
	[7] fine-tuned with ours	17.07	5.54	3.47	2.84
	Ours fine-tuned with [7]	22.86	6.74	**3.22**	**2.18**
Barrios et al. [8]	Li et al. [7]	10.51	5.66	3.29	2.05
	Ours	8.00	4.66	3.27	2.55
	[7] fine-tuned with ours	11.31	5.37	3.11	1.89
	Ours fine-tuned with [7]	**7.26**	**4.16**	**2.51**	**1.67**

**Table 5:** Evolution in results between training with our dataset with and without fine-tuning on Li et al.’s dataset [7]. The numbers correspond to the percentage of pixels that change category with the addition of fine-tuning. Green values under the diagonal show the pixels whose category is improved with fine-tuning, and red values above the diagonal show the pixels whose category is degraded with fine-tuning. The white values on the diagonal show the pixel for which there is no change of category.

Evolution between Training with Our Dataset only (Row) and Training with Fine-Tuning (Column)						
Li et al. [7]		0 ≤ err < 0.5	0.5 ≤ err < 1	1 ≤ error < 2	2 ≤ err < 4	err ≥ 4
Category improved	0 ≤ err < 0.5	64.65	7.21	1.22	0.18	0.17
15.82	0.5 ≤ err < 1	9.92	3.42	1.07	0.18	0.15
	1 ≤ err < 2	1.95	1.46	1.35	0.38	0.23
Category degraded	2 ≤ err < 4	0.26	0.25	0.48	0.57	0.36
11.15	err ≥ 4	0.32	0.27	0.4	0.51	3.05
Barrios et al. [7]		0 ≤ err < 0.5	0.5 ≤ err < 1	1 ≤ error < 2	2 ≤ err < 4	err ≥ 4
Category improved	0 ≤ err < 0.5	81.41	2.50	0.70	0.27	0.17
7.71	0.5 ≤ err < 1	4.13	1.50	0.70	0.24	0.12
	1 ≤ err < 2	0.73	0.67	0.92	0.41	0.21
Category degraded	2 ≤ err < 4	0.28	0.23	0.40	0.51	0.31
5.63	err ≥ 4	0.27	0.22	0.33	0.45	2.32

**Table 6:** Comparison of results between training with either our dataset and the one proposed in Li et al.’s work [7]. These comparisons of error are conducted for the small resolution and high-resolution disparity maps obtained by the neural network proposed by Barrios et al. [8]. Bold values have the lowest errors for their respective method and metric.

Output from [8]	Training Dataset	Bad 0.5	Bad 1	Bad 2	Bad 4
Results with a 3 × 3 configuration					
Small resolution (downsampled)	Li et al. [7]	16.39	11.37	8.42	6.30
	Ours	17.15	10.26	7.42	5.43
	[7] fine-tuned with ours	20.96	12.86	9.43	7.26
	Ours fine-tuned with [7]	**14.61**	**9.60**	**6.78**	**4.89**
High resolution (upsampled)	Li et al. [7]	16.60	11.46	8.22	5.75
	Ours	14.96	8.27	5.33	3.59
	[7] fine-tuned with ours	20.14	12.78	9.19	6.79
	Ours fine-tuned with [7]	**12.23**	**7.51**	**4.60**	**2.98**

**Table 7:** Comparison of quality of results between turning on or off fine-tuning of the refinement part on the method proposed by Barrios et al. [8].

Variant	Bad 0.5	Bad 1	Bad 2	Bad 4
No fine-tuning at all	14.96	8.27	5.33	3.59
Fine-tuning on every part	13.11	8.00	4.99	3.13
Fine-tuning except on the refinement part	12.23	7.51	4.60	2.98

## Data Availability

The training dataset, generated from a given set of free-to-use models and textures collected on various websites, and used to lead these experiments, is downloadable at https://revery.univ-reims.fr/dataset/big/Dataset_paper.tar (accessed on 21 December 2023).
